# Effects of half-dose spiomet treatment in girls with early puberty and accelerated bone maturation: a multicenter, randomized, placebo-controlled study protocol

**DOI:** 10.1186/s13063-022-07050-w

**Published:** 2023-01-24

**Authors:** Judit Bassols, Francis de Zegher, Marta Diaz, Gemma Carreras-Badosa, Cristina Garcia-Beltran, Elsa Puerto-Carranza, Cora Oliver-Vila, Paula Casano, Céline Alicia Franco, Rita Malpique, Abel López-Bermejo, Lourdes Ibáñez

**Affiliations:** 1 Maternal-Fetal Metabolic Research Group, Girona Biomedical Research Institute (IDIBGI), Girona, Spain; 2 Leuven Research & Development, University of Leuven, Leuven, Belgium; 3 Endocrinology Department, Pediatric Research Institute Sant Joan de Déu, University of Barcelona, Barcelona, Spain; 4 Centro de Investigación Biomédica en Red de Diabetes y Enfermedades Metabólicas Asociadas, Instituto de Salud Carlos III, Madrid, Spain; 5 Pediatric Endocrinology Research Group, Girona Biomedical Research Institute (IDIBGI), Girona, Spain; 6 Pediatrics, Dr. Josep Trueta Hospital, Girona, Spain; 7 Department of Medical Sciences, University of Girona, Girona, Spain

**Keywords:** Prenatal weight gain, Postnatal weight gain, Early puberty, Early menarche, PCOS, Ectopic fat, Bone maturation, Spironolactone, Pioglitazone, Metformin

## Abstract

**Background:**

A “mismatch” sequence of less prenatal weight gain and more postnatal weight gain may lead to ectopic lipid accumulation, and trigger the development of early adrenarche/pubarche and the activation of the gonadotropic axis resulting in early puberty and ending up in full-blown adolescent polycystic ovary syndrome (PCOS). In the present study, we assess whether a low-dose combination of generics that collectively reduce ectopic fat through different pathways can slow down the accelerated maturation in “mismatch” girls with early puberty.

**Methods:**

Randomized, placebo-controlled, multicenter, phase 2a, study in 64 girls [age, 8.0–9.3 years; birthweight (BW) for gestational age in lower tertile (−1.96< *Z*-score <−0.44), body mass index (BMI) in upper tertile (+0.44< *Z*-score < +1.96) and early progressive puberty (Tanner B2 at 7.7–9.0 years)]. Pharmacological intervention will be with a half-dose version of SPIOMET (mini-spiomet), a combination that reverts the PCOS phenotype in “mismatch” adolescents; mini-spiomet will contain spironolactone (25 mg/day, to raise brown adipose tissue activity), pioglitazone (3.75 mg/day, to raise adiponectin and insulin sensitivity), and metformin (425 mg/day, to raise AMPK activity and GDF15). Recruitment: 1 year; double-blind treatment: 1 year; open follow-up: 1 year; analyses and reporting: 1 year. Interventions: randomization (1:1) for placebo *vs* mini-spiomet. *Primary outcome*: annualized bone age advancement (0–1 year) by BoneXpert; *secondary outcomes*: insulin, IGF-I, high-molecular-weight adiponectin (HMW-adip), sex hormone binding globulin (SHBG), ultra-sensitive C-reactive protein (usCRP), androgens, luteinizing hormone (LH), follicle-stimulating hormone (FSH), oestradiol, growth-and-differentiation factor 15 (GDF15), C-X-C motif chemokine ligand-14 (CXCL14), safety parameters, and quantification of hepato-visceral fat.

**Discussion:**

The present study, if successful, may provide a first proof of the concept that the rapid maturation of girls with an upward mismatch between pre- and post-natal weight gain can be slowed down with a fixed low-dose combination of old and safe generics jointly targeting a reduction of ectopic fat without necessarily lowering body weight.

**Trial registration:**

EudraCT 2021-006766-21. Registered on May 30, 2022.

**Supplementary Information:**

The online version contains supplementary material available at 10.1186/s13063-022-07050-w.

## Administrative information

Note: the numbers in curly brackets in this protocol refer to SPIRIT checklist item numbers. The order of the items has been modified to group similar items (see http://www.equator-network.org/reporting-guidelines/spirit-2013-statement-defining-standard-protocol-items-for-clinical-trials/).

**Table:** 

Title {1}	Effects of half-dose spiomet treatment in girls with early puberty and accelerated bone maturation: a multicenter, randomized, placebo-controlled study protocol
Trial registration {2a and 2b}.	EudraCT: 2021-006766-21
Protocol version {3}	Version 3, 12/08/2022
Funding {4}	Carlos III Health Institute (ICI21/00005)
Author details {5a}	^1^Maternal-Fetal Metabolic Research Group, Girona Biomedical Research Institute (IDIBGI), Girona, Spain. ^2^Leuven Research & Development, University of Leuven, Belgium ^3^Endocrinology Department, Pediatric Research Institut Sant Joan de Déu, University of Barcelona, Barcelona, Spain. ^4^Centro de Investigación Biomédica en Red de Diabetes y Enfermedades Metabólicas Asociadas, Instituto de Salud Carlos III, Madrid, Spain ^5^Pediatric Endocrinology Research Group, Girona Biomedical Research Institute (IDIBGI), Girona, Spain ^6^Pediatrics, Dr. Josep Trueta Hospital, Girona, Spain ^7^Department of Medical Sciences, University of Girona, Girona, Spain
Name and contact information for the trial sponsor {5b}	NA
Role of sponsor {5c}	NA

## Introduction

### Background and rationale {6a}

Puberty underlines an important physical and psychosocial period of life where an individual develops secondary sexual characteristics and attains reproductive capacity. Puberty is signaled by the reactivation of the hypothalamic-pituitary-gonadal (HHG) axis, which triggers the release of kisspeptin and the onset of pulsatile hypothalamic gonadotrophin-releasing hormone (GnRH) secretion, which in turn drives pituitary gonadotrophin synthesis and downstream gonadal steroid secretion [1]. The precise mechanism triggering the reactivation of the HHG axis is not fully understood; however, GnRH neurosecretory activity and thus pubertal timing appear to be partly controlled by complex neuroendocrine pathways gathering genetic, nutritional, hormonal, metabolic, and environmental signals.

#### Adiposity and pubertal timing

Over the past decades, there has been a worldwide trend towards younger ages of pubertal onset and menarche in girls [2, 3]. Nowadays, mean age at puberty start is set up below age 10 years, representing an advancement of almost 3 months per decade from 1977 to 2013 [2]. In contemporary societies, the worldwide rise in childhood overweight/obesity appears to play a key role in the global decrease of the age at puberty. The pivotal role of nutrition and adiposity on pubertal timing has been known since nearly five decades ago, where Frisch and Revelle framed the “critical weight hypothesis” as a key determinant of pubertal start in girls [4]. Several cross-sectional and longitudinal studies have linked childhood adiposity with earlier pubertal onset, especially in girls [5, 6]. In some populations, the trend towards early puberty may be even more pronounced, in parallel with rapid gains of body weight [7], and has a sexual dimorphism, being more noticeable in girls [8]. This important rise cannot be explained by other environmental factors such as exposures to endocrine disruptors [9]. Also, a sudden weight gain over a short period of time associated to the lockdown for the coronavirus pandemic has been shown to associate to an increased incidence of precocious and accelerated puberty in Italian girls [10]. The concept that fat mass in childhood is linked to pubertal timing has recently been endorsed by longitudinal data from >2000 English girls, showing that more fat mass in childhood is followed by an earlier pubertal growth spurt and earlier pubertal completion [11]. Also, recent genome-wide association studies (GWAS) in humans have identified body mass index (BMI)-increasing alleles that associate with earlier age at menarche, pointing toward genetic co-regulation [12]. In addition, Mendelian randomization studies support a causal effect of increasing childhood BMI on the risk of early menarche (<12 years) [13]. Despite these strong epidemiologic and genetic links, the precise mechanism(s) underlying obesity-related early pubertal onset have remained elusive until the discovery of kisspeptin almost two decades ago, connecting the metabolic cues derived from adipose tissue and the regulation of GnRH secretion. It was shown that leptin, which has a permissive role in puberty onset, is able to up-regulate kisspeptin secretion in the hypothalamus, which in turn regulates the pulsatile secretion of GnRH [14]. Recently, a central ceramide signaling pathway has been unveiled as a novel mediator of obesity-induced early puberty in female rats [15]. Indeed, reduced signaling by ceramidase and also by AMP-activated protein kinase (AMPK) in the hypothalamus appears to link energy status and puberty-reproduction [15, 16]. Adiponectin — an adipokine with insulin-sensitizing and cardiovascular-protective properties — signals through its own transmembrane receptors to raise intracellular ceramidase activity, preventing the accumulation of unfavorable ceramide, for example, in the hypothalamus and in the liver [17].

#### Early puberty as an adaptive response to ectopic fat accumulation in “mismatch” girls

Earlier/faster maturation in girls has been hypothesized to be the clinical expression of an adaptive mechanism through which girls attempt to escape from ectopic lipid accumulation. This accumulation in turn, results from a mismatch between reduced prenatal weight gain (with reduced subcutaneous adipogenesis, and thus with a reduced capacity for safe lipid storage), and augmented postnatal weight gain (with augmented lipogenesis, and thus, an augmented need for lipid storage) [18]. Such a mismatch may lead to ectopic lipid accumulation, particularly in the liver and viscera (central obesity), the degree of which may be also influenced by (epi)genetic factors [19]. The endocrine expression of this mismatch tends to be the early development of insulin resistance, whereas its cardiovascular reflection is often a trend towards higher blood pressure starting in early childhood [18, 20–25]. There are close associations in childhood between the aforementioned mismatch and central fat and also between the mismatch and insulin resistance — as judged by homeostasis model assessment insulin resistance (HOMA-IR)- and between central fat and insulin resistance [24].

In prepubertal girls, the responses to central obesity include also a decrease in circulating sex hormone-binding globulin (SHBG) and adiponectin, which may be followed by an early and amplified adrenarche, with high levels of its marker, dehydroepiandrosterone-sulfate (DHEAS), and by the appearance of pubic (pubarche) and/or axillary hair, acne and pubertal odor before age 8 years [26–28]. These responses can be viewed as being adaptive since they result in accelerations of body growth and maturation that most likely represent a coordinated feedback mechanism to counteract ectopic adiposity [18]. If the ectopic lipid accumulation continues, then girls may develop another acceleration of growth and maturation by activating their gonadotrophic axis, conceivably again in a homeostatic attempt to escape from central adiposity. The reduced adiponectin concentrations may favor a reduced intracellular ceramidase activity and thus ceramide accumulation in the hypothalamus and liver triggering pubertal onset [15]. These novel insights may largely explain the worldwide trends towards younger ages at puberty start and menarche in girls [2, 3, 5, 11], which are known to associate to higher levels of delinquent and aggressive behavior and to more susceptibility to negative peer influences [29], and also to future risk of gestational diabetes [30], type 2 diabetes [31], and breast and endometrial cancer [32].

In girls with early puberty, the presence of a mismatch can be easily estimated by calculating the upward change in *Z*-score (or centile) between birthweight-for-gestational-age and BMI at onset of puberty. This “mismatch” hypothesis has now been tested in a cohort of girls with isolated variants of central precocious puberty from a single center in Paris [33], the majority of whom were found to have experienced an upward mismatch between prenatal and postnatal weight gain [34].

In the first years after menarche, when adult height is almost attained, the compensatory effect of body growth on central fat accumulation is lost. If the energy balance remains chronically positive, the underpinning drive of ectopic adiposity will also remain, and the endocrine-metabolic responses to this drive (insulin resistance, low adiponectin, and SHBG) will persist, and potentially result in a full-blown phenotype of adolescent polycystic ovary syndrome (PCOS) including luteinizing hormone (LH) hypersecretion which in turn, can drive ovarian androgen excess and oligo-anovulation [16].

#### Reduction of ectopic fat in “mismatch” girls with accelerated maturation and in adolescents with PCOS: pilot studies

Previous pilot studies performed by our group in rapidly maturing mismatch girls with precocious pubarche and/or early puberty have disclosed that metformin in monotherapy over a period of 3–4 years (up to 850 mg/day), can reduce central adiposity in viscera and liver [35–37], slow down bone maturation [38], delay pubertal onset [39], and decelerate the progression of puberty to menarche [40, 41] and to adolescent PCOS [42], while augmenting height gain [40, 41]. In mismatch adolescents with PCOS, a low-dose combination of spironolactone (50mg), pioglitazone (7.5mg), and metformin (850mg) in three separate tablets (SPIOMET) was recently shown to be capable of reversing the entire PCOS phenotype after only 1 year of treatment, including menstrual irregularities, hyperandrogenemia, and insulin resistance, through decreasing hepato-visceral fat excess [43–46]. Here, we propose to conduct a randomized, placebo-controlled, multicenter study using only half of the SPIOMET tablet, i.e., with half-dose spiomet (mini-spiomet: spironolactone 25mg; pioglitazone 3.75mg; metformin 425mg). Administering a triple combination instead of metformin in monotherapy will allow to decrease the dose of each component and to reduce the treatment period to 1 year. The rationale for using three different medications is that each of those medications targets a distinct mismatch-derived dysfunction.

*Spironolactone* is a steroidal aldosterone antagonist marketed as diuretic but serves as an anti-androgen at higher doses (up to 200 mg/day). Recently, it has been identified as a potent activator of brown adipose tissue (BAT), and thus as a potential driver of energy expenditure, and aims at fat repartitioning [47, 48]. Spironolactone was first approved in 1960, and it has been used for heart failure, and for other disorders (primary hyperaldosteronism, essential hypertension, edematous conditions). It is licensed for edema in the pediatric population in Europe at doses of approximately 3 mg/kg. No safety concerns related to the use of spironolactone have been raised since its approval [49]. In Europe and in the USA, spironolactone has been the anti-androgen of choice in the treatment of hirsutism for decades, with an excellent safety profile [50]. The only minor side effects reported at high dose (100 mg/day or more) are menstrual irregularities, and to a lesser extent, abdominal pain, polyuria, and dryness of the mouth [50]. There are essentially no safety concerns when dosed at only 25 mg/day (equal or less than 1 mg/kg/day), as will be in this study. Similarly, epidemiologic data show no evidence of an increased risk of any cancer associated with spironolactone use [51].

*Pioglitazone* is a thiazolidinedione (TZD) acting as an insulin sensitiser in adipose tissue, liver, and muscle. It raises circulating adiponectin, a driver of intracellular ceramidase [15, 52], and also insulin sensitivity via preferentially subcutaneous adipogenesis [53]. Pioglitazone was first approved in 1999, and in 2006, a fixed-dose combination containing pioglitazone and metformin was registered in Europe (Competact®, Glubrava®). At a low dose (7.5 mg/day), pioglitazone acts as an inhibitor of cyclin-dependent kinase 5 (CDK5)-mediated phosphorylation of peroxisome proliferator-activated receptor rather than as a peroxisome proliferator-activated receptor-gamma activator [54]. The use of pioglitazone has been questioned due to a purported higher risk for bladder cancer in older men with diabetes. A 10-year prospective study performed by the FDA to evaluate this connection concluded that it was non-existing [55]; accordingly, this association is considered to have been a “red herring” [56]. In adolescents with PCOS, low-dose pioglitazone (7.5 mg/day) has an excellent safety profile [43, 44]; pioglitazone appears to be well tolerated by children since no side effects were identified in children (with autism) receiving a 10-fold higher dose [57]. Pioglitazone is currently under investigation for a first pediatric indication within the SPIOMET context (see below). Spironolactone, when dosed at 50 mg/day in adults, is considered not to cause a clinically relevant drug-drug interaction with pioglitazone, via inhibition of hepatic CYP2C8, which is the main isoenzyme involved in pioglitazone’s metabolism [58]. In adipocytes, pioglitazone and spironolactone induce the expression of C-X-C motif chemokine ligand-14 (CXCL14), a chemokine that is released by BAT and protects against insulin resistance [48]. SPIOMET administration normalizes the low levels of CXCL14 in girls with PCOS, suggesting that CXCL14 may be among the mediators of SPIOMET’s benefits.

*Metformin* has pleiotropic effects but is generally considered to serve as a net “insulin sensitiser” in conditions of ectopic adiposity with insulin resistance; in addition, it raises AMPK activity, and the circulating concentrations of Growth-and-Differentiation Factor 15 (GDF15), a peptide hormone that reduces hepatic steatosis and raises intestinal glucose utilization thereby promoting weight loss [59–61]. Metformin was first approved in 1959, and since then several FDCs have been approved for the treatment of type 2 diabetes as first- and/or second-line therapies [62]. Worldwide, metformin is the drug most widely prescribed for the treatment of type 2 diabetes in adults and in children older than 10 years; its use has significantly increased in younger children and adolescents without diabetes, including for early maturation and PCOS in girls [37–41, 63]. Extensive experience has been gathered over the last 60 years related to the clinical use and safety of metformin [64]. In 2001, the European Medicines Agency (EMA) issued a favorable benefit-risk ratio for metformin that outlines its safety in humans [62]. The main side effects are gastrointestinal symptoms (~10%), that usually resolve after therapy start [64]. Lactic acidosis has been only described in cases of renal, cardiac and hepatic failure, or after intentional overdose [65]. A decrease of vitamin B12 serum levels may occur after long-term treatment but appears to be of no clinical relevance [66]. The combination of spironolactone and metformin is not associated with a higher incidence of adverse events compared to low-dose spironolactone or metformin in monotherapy [50]. Based on the EMA Summary of Product Characteristics (https://www.medicines.org.uk/emc/product/594/smpc#POSOLOGY), the dose range studied for metformin in clinical trials is mainly 200–850 mg/day, with a maximum of 2000 mg/day. Hence, the proposed dose of metformin (425 mg/day) will be in the lower recommended range, assuming that the weight of pubertal girls aged 8–9 years will be >25 kg [67].

#### Justification for the study

A mismatch sequence of reduced prenatal weight gain and augmented postnatal weight gain in girls may lead to ectopic lipid accumulation, particularly in the liver and viscera. Accelerated body growth and maturation, clinically expressed as early puberty, represents a concerted feedback mechanism to reduce such ectopic adiposity. We hypothesize that a reduction of ectopic adiposity with mini-spiomet (spironolactone 25 mg/day; pioglitazone 3.75 mg/day; metformin 425 mg/day) can slow down the rapid tempo of pubertal development and bone maturation. If this hypothesis is corroborated, then we intend to set up a multinational study wherein the hypothesis can be tested further, for example in other ethnic groups, as to extend the applicability of the present results.

### Objectives {7}

#### General

To ascertain whether a low-dose combination of generics within a single tablet known to collectively reduce ectopic adiposity through different pathways, can slow down the accelerated maturation in girls with early puberty and a “mismatch” sequence of less prenatal weight gain (resulting in a reduced subcutaneous adipogenesis and a reduced capacity for safe lipid storage), and more postnatal weight gain (resulting in more lipogenesis and more need for lipid storage).

#### Specific

1. To determine whether pharmacological intervention with a low-dose combination of spironolactone (25 mg/day), pioglitazone (3.75 mg/day), and metformin (425 mg/day) within a single tablet (mini-spiomet) over 1 year in “mismatch” girls with early puberty, reduces hepatic and visceral fat significantly more than placebo, as assessed by MRI.
2. To ascertain whether in girls treated with mini-spiomet, the reduction of hepatic and visceral fat associates to (1) a greater deceleration of bone maturation [as evaluated by Δ bone age (BA)/Δ chronological age (CA) ratio over 1 year]; (2) a slower pubertal *tempo* (as judged by the progression of Tanner stage); and (3) more normal levels of insulin, IGF-I, inflammation markers, sex steroids, HMW-adip, CXCL14, and GDF15, as compared to girls who received placebo.
3. To assess whether the benefits of mini-spiomet on hepato-visceral fat and endocrine-metabolic markers on-treatment are still detectable 1 year after treatment discontinuation.
4. To evaluate the tolerance and safety of mini-spiomet over 1 year (by assessing safety parameters), as well as the acceptance of the tablet (through a specific visual questionnaire).

### Trial design {8}

The mini-spiomet trial is designed as a double-blind, randomized, controlled, parallel-group, two-arm, superiority, multicenter phase 2a trial with 1:1 allocation ratio. Girls will be randomly assigned to receive daily either placebo (*n*=32) or a low-dose combination of three generics within a single tablet, namely mini-spiomet (spironolactone 25 mg + pioglitazone 3.75 mg + metformin 425 mg; *n*=32) for 1 year. After this year, all girls will be followed for 1 year. Halfway the subsequent year, all girls will be contacted for information on menarche.

Girls will be randomized (1:1) in blocks, according to age, birthweight (BW), and BMI. The study will test the effects of mini-spiomet (*vs* placebo) on bone maturation, endocrine-metabolic and inflammation variables and abdominal fat distribution, both on- and post-treatment.

## Methods: participants, interventions and outcomes

### Study setting {9}

The study subjects will be recruited in two tertiary centers in Spain: (1) Josep Trueta Hospital (HJT) and Institut d'Investigació Biomèdica de Girona Dr. Josep Trueta (IDIBGI) (Girona); (2) Sant Joan de Déu Hospital (HSJD) and Institut de Recerca Sant Joan de Déu (IRSJD) (Esplugues, Barcelona). Both centers are composed of (1) a referral hospital that provides an appropriate setting to examine patients and to conduct study visits and (2) a research institute with a laboratory that has the necessary equipment to perform the studies.

### Eligibility criteria {10}

Eligible participants will be girls (*n*=64), age 8.0–9.3 years, consecutively seen at the Pediatric Endocrinology Department from HJT (*n*=32) and HSJD (*n*=32) with a “mismatch” sequence of reduced prenatal weight gain and augmented postnatal weight gain followed by early, progressive puberty.

Inclusion criteria

1. Age at study start 8.0–9.3 years;
2. BW for gestational age in lower tertile (−1.96< *Z*-score < −0.44);
3. BMI for CA in upper tertile (+0.44< *Z*-score < +1.96) [2];
4. Early progressive puberty [bilateral breast development (Tanner stage 2)] starting between 7.7 and 9.0 years, with a minimum of 4 months of progression) [3, 4];
5. White ethnicity;
6. Full-term pregnancy: 37 ≤ gestational age < 42 weeks;
7. Height at 1st visit: 3rd percentile ≤ height ≤ 97th percentile; and
8. Written informed consent of parents or legal representative.

Exclusion criteria

1. Excessive delay or advance of bone age (more than 2 years for chronological age);
2. Tanner stage of breast development greater than 2;
3. Twin pregnancy;
4. Obesity at 1st visit (BMI *Z*-score above +1.96 for chronological age);
5. Evidence for a pathological cause of the rapid maturation (i.e., congenital adrenal hyperplasia due to 21-hydroxylase deficiency);
6. Known genetic abnormality or chronic conditions, including cardiovascular, neurological, immunological, metabolic, renal, endocrine, digestive, respiratory or oncological diseases;
7. Chronic use of medications, among others: anticoagulants, anti-inflammatories, oral hypoglycemic agents, antiandrogens, estrogens, progestins, glucocorticoids, digoxin. Only the use of paracetamol before or during the course of the study will be accepted;
8. Acute infections or intake of antibiotics or anti-inflammatory medication in the last 14 days;
9. Previous history of hypersensitivity to any of the drugs used in the clinical trial, or to its excipients; and
10. Any disease that, in the opinion of the investigator, compromises the inclusion of the subject in the clinical trial.

### Who will take informed consent? {26a}

Pediatric endocrinologists and/or research assistants involved in the trial will obtain written informed consent from at least one parent or legal guardian of participating girls.

### Additional consent provisions for collection and use of participant data and biological specimens {26b}

An additional written informed consent for biological sample collection will be obtained from at least one parent or legal guardian of participating girls.

## Interventions

### Explanation for the choice of comparators {6b}

This protocol is based on a pharmacological intervention with mini-spiomet, which is a half-dose version of SPIOMET which, in turn, is a combination that reverts the PCOS phenotype by reducing ectopic fat in “mismatch” adolescents. Mini-spiomet will contain spironolactone (25 mg/day, to raise brown adipose tissue activity), pioglitazone (3.75 mg/day, to raise adiponectin and insulin sensitivity), and metformin (425 mg/day, to raise AMPK activity and GDF15). We expect that these effects will collectively reduce ectopic fat and slow down the accelerated maturation in “mismatch” girls with early puberty. As there is no approved treatment for early puberty in girls, the comparator will be placebo.

### Intervention description {11a}

Participating girls will be randomly distributed into two subgroups (1:1):

- Mini-spiomet subgroup: girls will swallow a half-dose of the SPIOMET tablet once daily at dinner time, for 12 months. SPIOMET is a fixed-dose combination within a single tablet containing the active pharmaceutical ingredients spironolactone (50 mg), pioglitazone (7.5 mg), and metformin (850 mg), developed according to the formula that has been tested in a phase 2a trial in adolescents with PCOS [6] and is currently tested further in a multicenter, double-blind, placebo-controlled phase 2b trial that is funded through the EU’s Horizon 2020 programme (899671-SPIOMET4HEALTH) and is endorsed by the European Medicines Agency (EMA) via an approved Paediatric Investigation Plan (PIP; P/150/2021). The proposed excipients are povidone (polyvinylpirrolidone) k-30, microcrystalline cellulose, croscarmellose sodium, polyglykol 4000 PS, magnesium stearate and purified water.
- Placebo subgroup: girls will swallow a similar tablet (at similar timing and for a similar duration) containing only the aforementioned excipients.

The tablets containing placebo and those containing the active ingredients (SPIOMET) will have a score line to facilitate the split into two halves immediately before intake, will have the same size, shape and color, and will thus be indistinguishable from one another, to ensure the double-blind nature of the study.

### Criteria for discontinuing or modifying allocated interventions {11b}

In all participating girls, safety markers [blood count, circulating levels of alanine transaminase (ALT), aspartate transaminase (AST), gammaglutamyltransferase (GGT), thyroid-stimulating hormone (TSH), urea, creatinine, electrolyte panel, vitamin B12, folic acid] will be assessed at treatment start, after 6 months and 1 year on treatment, and after 1 year off treatment, as well as upon reporting any adverse events. Glucose blood levels <65 mg/dL and a progressive increase of creatinine, urea, transaminase or potassium levels, will be a reason for discontinuing the medication. In addition, the existence of signs and/or symptoms of hypoglycemia, the appearance of a persistent skin rash, abdominal pain, persistent nausea and/or vomiting, headaches, sinusitis, and severe bacterial infections, will be also a reason for discontinuation. In those cases, hepatic and renal function will be periodically monitored until the disappearance of the symptoms or the normalization of the altered parameters.

### Strategies to improve adherence to interventions {11c}

Participating girls will be contacted every 3 months — either in person within a medical visit or by phone — to maintain a close contact with them, and to monitor adherence and adverse events.

Adherence to treatment will be screened by history at each clinical visit and by tablet counts in the pharmacy dispensing the study medications (ratio between the number of tablets prescribed and dispensed for the period between two hospital appointments and the number of half tablets returned by the patient at the following appointment).

### Relevant concomitant care permitted or prohibited during the trial {11d}

Girls who participate in this trial cannot participate in another trial at the same time.

Permitted concomitant treatments:

- Acetaminophen.

Prohibited concomitant treatments:

- Anticoagulants, anti-inflammatory medications, (other) oral hypoglycemic drugs, (other) anti-androgens, estrogens, progestogens, digoxin;
- Potassium-sparing diuretics, as well as supplements or food complements based on it;
- Oral contraceptives throughout the duration of the study;
- Iodinated contrast agents; and
- Caution should be taken with the concomitant use of drugs that can cause hyperkalemia or metabolic acidosis, such as non-steroidal anti-inflammatory drugs, including selective cyclooxygenase II inhibitors, angiotensin-converting enzyme inhibitors, receptor antagonists of angiotensin II and diuretics, especially loop diuretics.

### Provisions for post-trial care {30}

Each of the participating centers holds a specific insurance for patients participating in clinical trials.

### Outcomes {12}

#### Primary outcome

The primary outcome is annualized bone age (BA) advancement over the on-treatment year. A hand and wrist X-ray of the left hand will be obtained at the treatment start and after 1 year on treatment. BA advancement will be measured by an automated/blinded method called BoneXpert (Visiana, Denmark) [1].

#### Secondary outcomes

- ◦ Clinical variables: weight, height, BMI, waist and hip circumference and their ratio (WHR), systolic blood pressure (SBP), diastolic blood pressure (DBP), and Tanner stage [4].
- ◦ Endocrine-metabolic variables:
  - fasting glucose, insulin, HOMA-IR [5], IGF-I
  - LH, FSH, testosterone, androstenedione, SHBG, FAI and estradiol
  - triglycerides; total, low- and high-density lipoprotein (LDL and HDL) cholesterol
  - ultra-sensitive C-reactive protein (usCRP), GDF-15, HMW-adip, CXCL14.
- ◦ Safety markers: blood count, circulating concentrations of alanine transaminase (ALT), aspartate transaminase (AST), gammaglutamyltransferase (GGT), thyroid-stimulating hormone (TSH), urea, creatinine, electrolyte panel, vitamin B12, folic acid.
- ◦ Abdominal fat partitioning (subcutaneous visceral area) and intrahepatic fat by MRI.
- ◦ Dietary habits, tablet acceptability, study adherence, adverse events.

### Participant timeline {13}

**Fig. 1:**
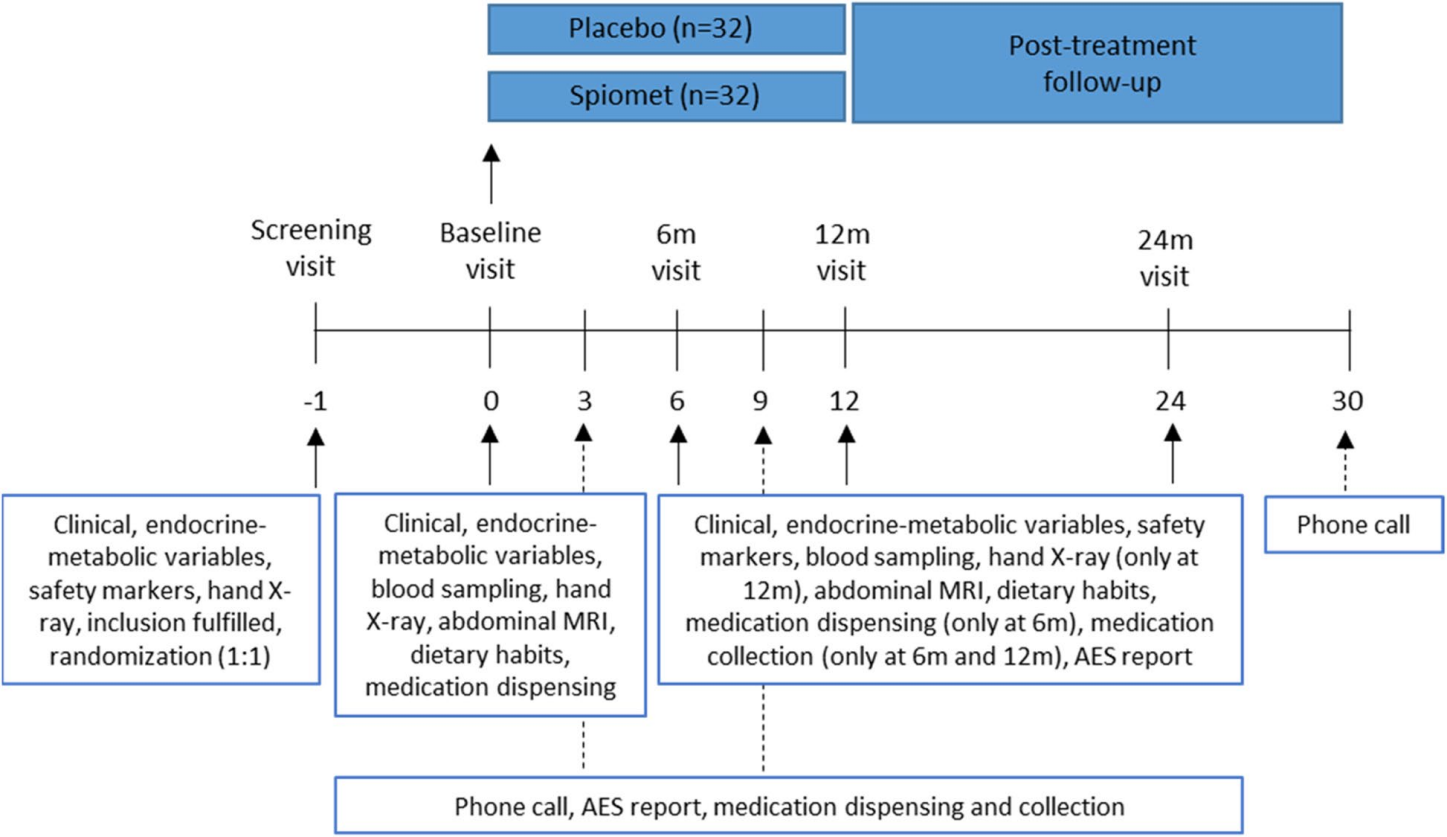
Diagram of the study design

Figure 1 depicts the diagram of the study design.

### Sample size {14}

In a previous study performed in girls with a history of low-BW and rapid postnatal catch-up in weight, metformin treatment for 4 years (425 mg for 2 years, then 850 mg for 2 years) slowed down the accelerated bone maturation [38]. The progression of bone maturation, as assessed by the mean ratio of Δ BA over Δ CA, was faster in untreated girls [≈1.20 ± 0.2 (SD)] as compared to metformin-treated girls [≈1.00 ± 0.2 (SD)] (*P*≤0.05). To obtain a significance level of 0.05% and power of 90%, a total of 42 girls (21 in each study arm) must be included to detect the minimal relevant difference in bone age progression (1.20 vs 1.00 years) (https://clincalc.com/stats/samplesize.aspx). The number of patients has been further increased (*n*=32 in each study arm) to guarantee the necessary sample size, in the case up to one third of the participants drop out of the study.

### Recruitment {15}

Candidate girls will be recruited at the Outpatient Clinic of HJT and HSJD among those referred by pediatricians of primary care for early onset of puberty. Confirmation of eligibility will require a clinical visit where inclusion and exclusion criteria will be carefully screened. A baseline blood test will be performed to assess circulating concentrations of 17-hydroxyprogesterone (17-OHP) in order to rule out congenital adrenal hyperplasia due to 21-hydroxylase deficiency or abnormal thyroid, kidney, or liver function, all of which are exclusion criteria. If a girl meets all the inclusion criteria and none of the exclusion criteria, she will become eligible to participate in the study, the objective of which will then be explained so that written informed consent can be obtained from at least one parent or legal guardian.

## Assignment of interventions: allocation

### Sequence generation {16a}

Patients will be randomized using Stratified Permuted Block Randomization. Trial participants will be subdivided into strata, and then permuted block randomization will be used for each stratum. A block randomization list with 4 randomization blocks will be created. Each block will include 8 patients. There will be a randomization list per center and the total number of subjects per center will be 32 patients (4 blocks x 8 patients/block) with a final distribution of 16 patients who will receive mini-spiomet and 16 who will receive placebo at each center.

BLOCK 1: Medication: mini-spiomet vs placebo

BLOCK 2: Chronological age (CA): 7.7≤ CA≤8.4 years vs 8.5 ≤ CA ≤9.3 years

BLOCK 3: Birth weight (BW-SDS, in standard deviations by sex and gestational age):

−1.96 (3rd percentile) ≤BW-SDS<−1.036 (15th percentile)
−1.036 (15th percentile) ≤BW-SDS ≤ −0.44 (33rd percentile)

BLOCK 4: Body mass index (BMI-SDS, in standard deviations by sex and chronological age at the clinical visit):

- +0.44 (66th percentile) ≤ BMI-SDS ≤ +1.036 (85th percentile)
- 1.036 (85th percentile) < BMI-SDS ≤ 1.96 (97th percentile)

### Concealment mechanism {16b}

A randomization list per center will be generated using the program Blockrand: Randomization for Block Random Clinical Trials R package; version 1.5; Published: 2020; Author: Gdreg Snow; https://CRAN.R-project.org/package=blockrand. Patients will be sequentially allocated to a group by the pharmacy service of each center.

### Implementation {16c}

Upon receipt of informed consent (see above), study participants will be enrolled by the recruiting investigator, usually a pediatric endocrinologist, and will be assigned a center-specific registration number that will appear as HSJD-miniSPIOMET-number or HJT-miniSPIOMET-number; this unique number will be used to identify each participant throughout the study. Subsequently, the hospital pharmacy will receive the information that will allow for randomization and for delivery of the allocated medication.

## Assignment of interventions: blinding

### Who will be blinded {17a}

Throughout the on-treatment phase, the pharmacy at each center will be the sole entity with knowledge on treatment allocation.

After locking the on-treatment data base, the statistically involved investigators will be unblinded in order to allow for data analysis. The statistical analyses will be performed by independent investigators who will have no contact with the patient or with the clinical investigators.

Study participants and clinically involved investigators will remain blinded until the end of the post-treatment year.

### Procedure for unblinding if needed {17b}

If a girl/family wishes to withdraw from the trial or if an adverse event leads to withdrawal from the study, then the family will be invited to an end-of-study visit which will be followed by disclosure of treatment allocation. The girl will continue to receive standard care.

## Data collection and management

### Plans for assessment and collection of outcomes {18a}

#### Collection of outcomes

*Baseline evaluation (time 0):* will include the clinical visit to collect clinical variables: height, weight, BMI, Tanner stage, waist and hip circumference and their ratio (WHR), SBP, DBP, a hand and wrist X-ray (to assess bone age), MRI (to assess abdominal fat partitioning and hepatic fat) and blood sampling in the fasting state (to assess endocrine-metabolic and inflammation variables and safety markers). Dietary habits will be also recorded through a standard questionnaire (Kidmed).

*Evaluation on treatment (time 6 months and 1 year):* a clinical visit with the same parameters collected at baseline, an abdominal MRI, and a blood sample extraction (to assess endocrine-metabolic and inflammatory variables and safety markers) will be performed after 6 months and after 1 year on treatment. Dietary habits, study adherence, acceptability of the tablet, and adverse events will be also recorded. After 1 year of treatment, a hand and wrist X-ray will be performed for bone age assessment.

*Evaluation post-treatment (time 2 years):* will include a clinical visit 1 year after treatment discontinuation, collecting the same parameters as at time 0, 6 months, and 1 year; in addition, an abdominal MRI scan and a blood sampling (to assess endocrine-metabolic and inflammation variables and safety markers) will be performed. Dietary habits, study adherence, acceptability of the tablet, and adverse events will be also recorded. Six months later a phone call will be made to ascertain the presence or absence of menarche.

#### Assessment of outcomes

*Bone age:* A hand and wrist X-ray of the left hand will be taken at the Radiology Department either at HJT or HSJD, at treatment start and after 1 year on treatment. At each center, bone age will be determined with the same BoneXpert programme [version 3; February 2021 (Visiana, Denmark)] [38]. This method will yield bone ages by Greulich & Pyle (GP) and by Tanner-Whitehouse 2 (TW2). Bone age maturation will be annualized by calculating the ratio between the increase of BA over the increase in CA (ΔBA/ ΔCA) [38].

*Clinical characterization:* Clinical variables will be obtained during the clinical visits with the pediatric endocrinologist at HJT and HSJD. The same investigator at each center will assess the clinical variables at times 0, 6 months, 1 year, and 2 years. Halfway the second post-treatment year, the girls will be contacted by phone to discuss the absence or timing of menarche.

*Blood sampling:* 23 mL of peripheral blood in a fasting state will be drawn in the early morning at each clinical center, at baseline (time 0), after 6 months on treatment, after 1 year on treatment, and 1 year off treatment. Blood samples will be stored (at −80 °C) at the Pediatric Endocrinology lab (IDIBGI) and at the Metabolic Endocrinology lab (IRSJD) until analyses.

*Endocrine-metabolic variables analysis:*

- The clinical laboratory at both HJT and HSJD will be responsible for analyzing (1) glucose (by the glucose oxidase method); (2) fasting insulin, IGF-I, SHBG, and usCRP (by immuno-chemiluminescence); (3) LH, FSH, and oestradiol (by immuno-chemiluminescence); (4) total cholesterol, LDL-cholesterol, HDL-cholesterol, and triglycerides (by molecular absorption spectrometry).
- Circulating testosterone and androstenedione concentrations will be centrally assessed at the Bioanalysis Research lab [Institut Hospital del Mar d'Investigacions Mèdiques (IMIM), Hospital del Mar] by liquid chromatography-tandem mass spectrometry. The free androgen index (FAI) will be calculated with the following formula: testosterone (nmol/L) × 100/SHBG (nmol/L).
- The analysis of circulating HMW-adip, GDF-15, and CXCL14 concentrations will be centralized at the Metabolic Endocrinology laboratory (IRSJD). Circulating levels of HMW-adip will be analyzed by ELISA (Millipore, St Louis, MO), with intra- and inter-assay coefficients of variation (CVs) <9%. Serum GDF-15 will be assessed using a specific human ELISA kit (R&D Systems, Minneapolis) with intra- and inter-assay CVs <6%. Serum CXCL14 will be assessed using a specific ELISA kit (Ray Biotech, Norcross, GA, USA), with a sensitivity of 0.7 ng/ ml, and inter- and intra- assay CV less than 12%.

*Safety markers analysis:* The clinical laboratories at HJT and HSJD will analyze (1) blood count (by flow cytometry and colorimetric assay); (2) circulating levels of ALT, AST, GGT, TSH, urea, electrolyte panel, and creatinine (by molecular absorption spectrometry); and (3) vitamin B12 and folic acid (by immuno-chemiluminescence).

*Abdominal and hepatic fat assessment:* Abdominal fat distribution *(subcutaneous and visceral)* and intrahepatic fat will be analyzed by MRI at both clinical sites with the same scan [multiple slice 1.5 Tesla scan (Signa LX Echo Speed Plus Excite, General Electric Healthcare, Milwaukee, WI)]. The assessments will be performed at Clínica Girona (Girona) and CETIR Medical Centre (Barcelona). Scans will be performed by the same operator at each center, blinded to the treatment allocation, at times 0, 6 months, 1 year, and 2 years. A central reader blinded to treatment allocation will be appointed to process and interpret the images from both study centers (QUIBIM, Valencia, Spain).

*Dietary habits:* The quality of the dietary habits will be assessed by a modified version of the Kidmed questionnaire. Girls will complete the test during the clinical visits at the corresponding center (at times 0, 6 months, 1 year and 2 years).

*Acceptability of the tablet:* The patient's opinion regarding the palatability and swallowability of the tablet will be recorded by using a simple questionnaire with numerical rating scales and the integrated 100 mm visual analog scale (VAS)/facial hedonic scale [3, 4]. Patients will answer the questionnaire during the clinical visits at each center at times 6 months and 1 year.

All outcomes will be measured as previously described (domain, specific measure, and time points), stated as mean values, and compared between groups at the specific time points.

**Fig. 2:**
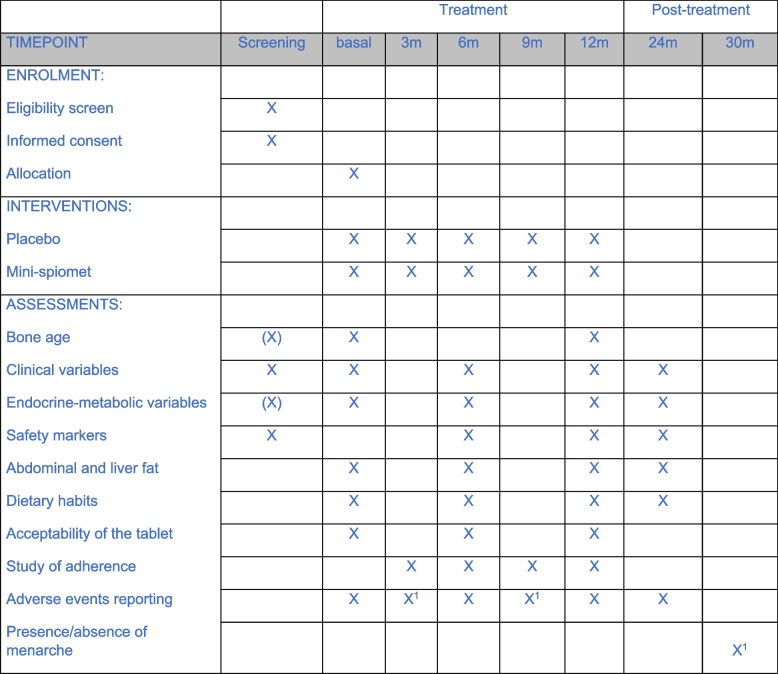
Schedule of enrolment, interventions, and assessments. ^1^ Phone visit; (X) will be performed in case it has not been done within the period of 3 weeks prior to the start of the medication

Figure 2 depicts the schedule of enrolment, interventions, and assessments.

### Plans to promote participant retention and complete follow-up {18b}

Patients will be contacted every 3 months, either in person with a medical visit or by phone call, in order to maintain a close contact with the patient as to ensure treatment compliance and reduce the risk of loss to follow-up.

### Data management {19}

The study will collect three types of data:

- Clinical data: will consist of numerical data that will be obtained by the staff during the clinical visit or after blood tests (that will be downloaded from the clinical history as a .pdf file)
- Imaging data: will consist of images (.tiff or .psd files) that will be obtained by MRI and X-ray and that will be analyzed with the corresponding program software.
- Information from patient questionnaires (Kidmed nutrition questionnaire)

Patient data will be recorded in anonymized form (without reference to the patient’s name) using exclusively the patient's identification code. Patient identification code lists will remain at the recruiting sites and will be stored separately from the trial data. These data will be only used for the re-identification of the patients, if needed.

The research data of this project will be stored in a REDCap database. The data will be collected and entered in the electronic database by the members of the research group (using a username and password). The consistency and quality of the data will be controlled to ensure that no data are accidentally changed and the accuracy of data is maintained over its entire life cycle.

At the end of the project, data will be recovered from REDCap and an SPSS file (.sav) will be created to perform the statistical analysis of the results. No sensitive data will be present in this file.

### Confidentiality {27}

All data of this trial will be anonymized and recorded on the hospital’s servers. No sensitive data will be externally available.

### Plans for collection, laboratory evaluation, and storage of biological specimens for genetic or molecular analysis in this trial/future use {33}

Blood samples will be processed and stored (at −80 °C) at the Pediatric Endocrinology lab (IDIBGI) and at the Metabolic Endocrinology lab (IRSJD) until analysis. The remnant samples will be kept in a private collection with registration code C0007072 (Instituto de Salud Carlos III; Madrid) to be used in future studies related to this research line.

## Statistical methods

### Statistical methods for primary and secondary outcomes {20a}

The statistical analyses will be performed with the SPSS program, version 23.0 (SPSS, Chicago, Illinois, USA). The analysis of quantitative variables of independent groups will be performed using ANOVA for repeated measurements in models adjusted for potential confounding variables. The association between BA, clinical variables, endocrine-metabolic, and imaging variables will be sought by correlation and multiple regression analysis. A statistical analysis will be done by intention to treat and another with the patients who have completed follow-up. *p*<0.05 will be considered statistically significant.

### Interim analyses {21b}

No interim analysis will be done. Statistical analysis will be done once the trial has been finished.

### Methods for additional analyses (e.g., subgroup analyses) {20b}

No subgroup analysis will be done.

### Methods in analysis to handle protocol non-adherence and any statistical methods to handle missing data {20c}

An intention-to-treat analysis will be performed including all participants randomized to either placebo or mini-spiomet, ignoring non-compliance, protocol deviations, withdrawal, and anything that happened after randomization. If necessary, for the withdrawn participants, the missing data will be filled with the data of previous visits.

### Plans to give access to the full protocol, participant-level data and statistical code {31c}

The full protocol, participant-level dataset, and statistical code will be provided on demand.

## Oversight and monitoring

### Composition of the coordinating center and trial steering committee {5d}

This is a multicenter study that will be held in (1) Josep Trueta Hospital (HJT) and Institut d'Investigació Biomèdica de Girona Dr. Josep Trueta (IDIBGI) (Girona) and (2) Sant Joan de Déu Hospital (HSJD) and Institut de Recerca Sant Joan de Déu (IRSJD) (Esplugues, Barcelona). The coordinating center is HJT-IDIBGI. The trial steering committee will be composed by the principal investigator of each institution (A.L-B and L.I) and the research coordinators (J.B and R.M) and will meet once per semester to coordinate and supervise all the tasks. A group of nurses, pediatricians, and researchers at each institution will provide day-to-day support for the trial.

### Composition of the data monitoring committee, its role and reporting structure {21a}

ADKNOMA Health Research SL is a Clinical Research Operator (CRO) that will provide support during the trial development process as a clinical regulatory specialist and will be responsible of the trial monitoring and data audit, regulatory issues, pharmacovigilance, and coordination of security activities.

### Adverse event reporting and harms {22}

The nature, severity, treatment, and outcome of any potential adverse event will be recorded by the clinical investigator seeing the patient at each center during the clinical visits (at 6 months, 1 year, and 2 years) or at the phone call contacts (at 3 and 9 months).

All adverse events and harms, whether expected or unexpected, will be collected in a non-systematically way asking the patient about their health, and will be coded according to MedDRA (CTCAE v4).

### Frequency and plans for auditing trial conduct {23}

The CRO ADKNOMA will be responsible for the auditing of the trial. A minimum of 6 monitoring visits are expected to be performed during the study. Annual reports will be provided.

### Plans for communicating important protocol amendments to relevant parties (e.g., trial participants, ethical committees) {25}

Protocol modifications will be approved by the ethical committees of the participant centers and by the AEMPs agency, and will be communicated to trial investigators.

### Dissemination plans {31a}

The results of the project will be disseminated at the scientific level (articles in peer-reviewed journals; presentations at national and international meetings) and at the society level (press releases, informative talks, web, radio, television).

## Discussion

### Innovation and originality

Early puberty is a prevalent entity and is the second reason for consultation in pediatric endocrinology clinics. The trend has significantly increased in the last decade, concomitantly with the increase of overweight/obesity in children. Early puberty may result in psychosocial maladjustment and risky behaviors in adolescence and may associate with several co-morbidities in adulthood. The proposed novel treatment with a low-dose combination of generics (mini-spiomet) is the first to target the pathophysiological roots of the entity, and therefore to hold the potential of reducing short- and long-term co-morbidities, and thus to have a beneficial impact on the economic burden of health care systems.

### Strengths and limitations

The main strengths are the double-blind, placebo-controlled design of the study, the assessment of bone maturation by an automated method, and the longitudinal assessment of abdominal fat partitioning and of novel markers of brown adipose tissue and insulin sensitization. The main limitation is the lack of ethnic variability in the present study population.

## Trial status

Protocol version 3; 12-08-22. Expected start date: November 2022. Expected completion date: March 2026.

## Supplementary Information

**Additional file 1: Supplementary Table.** WHO Trial Registration Data Set.

## Data Availability

The study investigators will have access to the final dataset, and they will not have any contractual agreement that limits such access.

## Abbreviations

ALT: Alanine transaminase
AMPK: AMP-activated protein kinase
AST: Aspartate transaminase
BA: Bone age
BAT: Brown adipose tissue
BW: Birthweight
BMI: Body mass index
CA: Chronological age
CXCL14: C-X-C motif chemokine ligand-14
DBP: Diastolic blood pressure
DHEAS: Dehydroepiandrosterone-sulfate
FAI: Free androgen index
FSH: Follicle-stimulating hormone
GDF15: Growth-and-differentiation factor 15
GGT: Gammaglutamyltransferase
GnRH: Gonadotrophin-releasing hormone
GWAS: Genome-wide association studies
HDL: High-density lipoprotein
HHG: Hypothalamic-pituitary-gonadal
HJT: Josep Trueta Hospital
HMW-adip: High-molecular-weight adiponectin
HOMA-IR: Homeostasis model assessment insulin resistance
HSJD: Sant Joan de Déu Hospital
IDIBGI: Institut d'Investigació Biomèdica de Girona Dr. Josep Trueta
IGF-I: Insulin growth factor 1
IRSJD: Institut de Recerca Sant Joan de Déu
LDL: Low-density lipoprotein
LH: Luteinizing hormone
MRI: Magnetic resonance imaging
PCOS: Polycystic ovary syndrome
SBP: Systolic blood pressure
SD: Standard deviation
SHBG: Sex hormone binding globulin
TSH: Thyroid-stimulating hormone
TZD: Thiazolidinedione
usCRP: Ultra-sensitive C-reactive protein
WHR: Waist-hip ratio
