# Correction: Effects of half-dose spiomet treatment in girls with early puberty and accelerated bone maturation: a multicenter, randomized, placebo-controlled study protocol

**DOI:** 10.1186/s13063-023-07483-x

**Published:** 2023-10-03

**Authors:** Judit Bassols, Francis de Zegher, Marta Diaz, Gemma Carreras‑Badosa, Cristina Garcia‑Beltran, Elsa Puerto‑Carranza, Cora Oliver‑Vila, Paula Casano, Celine Alicia Franco, Rita Malpique, Abel Lopez‑Bermejo, Lourdes Ibanez

**Affiliations:** 1 Maternal‑Fetal Metabolic Research Group, Girona Biomedical Research Institute (IDIBGI), Girona, Spain; 2 Leuven Research & Development, University of Leuven, Leuven, Belgium; 3 Endocrinology Department, Pediatric Research Institute Sant Joan de Deu, University of Barcelona, Barcelona, Spain; 4 Centro de Investigacion Biomedica en Red de Diabetes Y Enfermedades Metabolicas Asociadas, Instituto de Salud Carlos III, Madrid, Spain; 5 Pediatric Endocrinology Research Group, Girona Biomedical Research Institute (IDIBGI), Girona, Spain; 6 Pediatrics, Dr. Josep Trueta Hospital, Girona, Spain; 7 Department of Medical Sciences, University of Girona, Girona, Spain

**Correction: Trials 24, 56 (2023)**

**https://doi.org/10.1186/s13063-022-07050-w**

After publication of the article [1] the authors noticed several errors related to the inclusion and exclusion criteria. The clinical trial registration has been updated and the ethical committee has reviewed the changes.

**Table:** 

**Incorrect**	Correct
Inclusion criteria 1) Age at study start 8.0–9.3 years; 2) BW for gestational age in lower tertile (− 1.96 < Z-score < − 0.44); 3) BMI for CA in upper tertile (+ 0.44 < Z-score < + 1.96) [2]; 4) Early progressive puberty [bilateral breast development (Tanner stage 2)] starting between 7.7 and 9.0 years, with a minimum of 4 months of progression) [3, 4]; 5) White ethnicity; 6) Full-term pregnancy: 37 ≤ gestational age < 42 weeks; 7) Height at 1st visit: 3rd percentile ≤ height ≤ 97th percentile; and 8) Written informed consent of parents or legal representative.	*Inclusion criteria* 1) Age at study start 8.0–9.3 years; 2) BW for gestational age in lower tertile (-2.5 < Z-score < 0); 3) BMI for CA in upper tertile (0 < Z-score < + 2.5) (2); 4) Early progressive puberty [bilateral breast development (Tanner stage 2)] starting between 7.7- 9.3 years, with a minimum of 2 months of progression) (3,4); 5) White ethnicity; 6) Full-term or late preterm pregnancy: 34 ≤ gestational age < 42 weeks; 7) Height at 1st visit: 3rd percentile ≤ height ≤ 97th percentile (adjusted by pubertal stage); 8) Written informed consent of parents or legal representative.
*Exclusion criteria* 1) Excessive delay or advance of bone age (more than 2 years for chronological age); 2) Tanner stage of breast development greater than 2; 3) Twin pregnancy; 4) Obesity at 1st visit (BMI Z-score above + 1.96 for chronological age); 5) Evidence for a pathological cause of the rapid maturation (i.e., congenital adrenal hyperplasia due to 21-hydroxylase deficiency); 6) Known genetic abnormality or chronic conditions, including cardiovascular, neurological, immunological, metabolic, renal, endocrine, digestive, respiratory or oncological diseases; 7) Chronic use of medications, among others: anticoagulants, anti-inflammatories, oral hypoglycemic agents, antiandrogens, estrogens, progestins, glucocorticoids, digoxin. Only the use of paracetamol before or during the course of the study will be accepted; 8) Acute infections or intake of antibiotics or anti-inflammatory medication in the last 14 days; 9) Previous history of hypersensitivity to any of the drugs used in the clinical trial, or to its excipients; and 10) Any disease that, in the opinion of the investigator, compromises the inclusion of the subject in the clinical trial.	*Exclusion criteria* 1) Excessive delay or advance of bone age (more than 2 years for chronological age); 2) Tanner stage of breast development greater than 2; 3) Twin pregnancy; 4) Obesity at 1st visit (BMI Z-score above + 2.5 for chronological age); 5) Evidence for a pathological cause of the rapid maturation (i.e., congenital adrenal hyperplasia due to 21-hydroxylase deficiency); 6) Known genetic abnormality or chronic conditions, including cardiovascular, neurological, immunological, metabolic, renal, endocrine, digestive, respiratory or oncological diseases; 7) Chronic use of medications, among others: anticoagulants, anti-inflammatories, oral hypoglycemic agents, antiandrogens, oestrogens, progestins, glucocorticoids, digoxin. Only the use of paracetamol before or during the course of the study will be accepted; 8) Acute infections or intake of antibiotics or anti-inflammatory medication in the last 14 days; 9) Previous history of hypersensitivity to any of the drugs used in the clinical trial, or to its excipients; 10) Any disease that, in the opinion of the investigator, compromises the inclusion of the subject in the clinical trial.
