# Supplementary material for: Effects of half-dose spiomet treatment in girls with early puberty and accelerated bone maturation: a multicenter, randomized, placebo-controlled study protocol
Source: Trials. 2023 Jan 24;24:56. doi: 10.1186/s13063-022-07050-w (PMC9873221; doi:10.1186/s13063-022-07050-w)
Supplement: Supplementary file 1 — Additional file 1: Supplementary Table. WHO Trial Registration Data Set. [file 13063_2022_7050_MOESM1_ESM.docx]

**Supplementary Table**: WHO Trial Registration Data Set

| **Data category** | **Information** |
| --- | --- |
| Primary registry and trial identifying number | EudraCT  2021-006766-21 |
| Date of registration in primary registry | 30 May, 2022 |
| Source(s) of monetary or material support | Carlos III Health Institute (ICI21/00005) |
| Contact for public queries | Girona Biomedical Research Institute (IDIBGI) [872 987 087] |
| Contact for scientific queries | Dr. Lourdes Ibáñez, Pediatric Research Institut Sant Joan de Déu, Barcelona, Spain [[lourdes.ibanez@sjd.es](mailto:lourdes.ibanez@sjd.es)]  Dr. Abel López-Bermejo, Dr. Josep Trueta Hospital, Girona, Spain [alopezbermejo@idibgi.org] |
| Public title | MINI-SPIOMET |
| Scientific title | Towards a treatment for accelerated maturation in girls testing spiomet in a randomised placebo controlled, multicentre study |
| Countries of recruitment | Spain |
| Health condition(s) or problem(s) studied | Girls with advanced puberty and accelerated bone maturation |
| Intervention(s) | Active comparator: mini-spiomet (spironolactone 25 mg/d, pioglitazone 3.75 mg/d, and metformin 425 mg/d per day)  Placebo comparator: excipients (povidone k-30, microcrystalline cellulose, croscarmellose sodium, polyglykol 4000 PS, magnesium stearate and purified water) |
| Key inclusion and exclusion criteria | *Inclusion criteria*  1) Age at study start 8.0-9.3 years;  2) BW for gestational age in lower tertile (-1.96< Z-score < -0.44);  3) BMI for CA in upper tertile (+0.44< Z-score < +1.96);  4) Early progressive puberty [bilateral breast development (Tanner stage 2)] starting between 7.7- 9.0 years, with a minimum of 4 months of progression);  5) White ethnicity;  6) Full-term pregnancy: 37 ≤ gestational age < 42 weeks;  7) Height at 1st visit: 3rd percentile ≤ height ≤ 97th percentile;  8) Written informed consent of parents or legal representative.  *Exclusion criteria*  1) Excessive delay or advance of bone age (more than 2 years for chronological age);  2) Tanner stage of breast development greater than 2;  3) Twin pregnancy;  4) Obesity at 1st visit (BMI Z-score above +1.96 for chronological age);  5) Evidence for a pathological cause of the rapid maturation (i.e., congenital adrenal hyperplasia due to 21-hydroxylase deficiency);  6) Known genetic abnormality or chronic conditions, including cardiovascular, neurological, immunological, metabolic, renal, endocrine, digestive, respiratory or oncological diseases;  7) Chronic use of medications, among others: anticoagulants, anti-inflammatories, oral hypoglycemic agents, antiandrogens, oestrogens, progestins, glucocorticoids, digoxin. Only the use of paracetamol before or during the course of the study will be accepted;  8) Acute infections or intake of antibiotics or anti-inflammatory medication in the last 14 days;  9) Previous history of hypersensitivity to any of the drugs used in the clinical trial, or to its excipients;  10) Any disease that, in the opinion of the investigator, compromises the inclusion of the subject in the clinical trial. |
| Study type | Interventional Allocation: randomized Intervention model: parallel assignment Masking: double blind  Primary purpose: slow down the accelerated maturation in “mismatch” girls with early puberty Phase 2a |
| Target sample size | 64 |
| Recruitment status | Recruiting |
| Primary outcome(s) | Annualised bone age advancement (0-1 yr) |
| Key secondary outcomes | Clinical variables: weight, height, BMI, waist and hip circumference and their ratio (WHR), systolic blood pressure (SBP), diastolic blood pressure (DBP), and Tanner stage  Clinical variables: weight, height, BMI, waist and hip circumference and their ratio (WHR), systolic blood pressure (SBP), diastolic blood pressure (DBP), and Tanner stage (4).  Endocrine-metabolic variables: fasting glucose, insulin, HOMA-IR, IGF-I, LH, FSH, testosterone, androstenedione, SHBG, FAI and estradiol, triglycerides; total, low- and high-density lipoprotein (LDL and HDL) cholesterol, ultra-sensitive C-reactive protein (usCRP), GDF-15, HMW-adip, CXCL14.  Safety markers: blood count, circulating concentrations of alanine transaminase (ALT), aspartate transaminase (AST), gammaglutamyltransferase (GGT), thyroid-stimulating hormone (TSH), urea, creatinine, electrolyte panel, vitamin B12, folic acid.  Abdominal fat partitioning (subcutaneous visceral area) and intrahepatic fat |
